# Carbon pricing and aggregate macroeconomic performance in the Eurozone: a contribution to the climate policy debate using the EU ETS and Macroeconomic Performance Index

**DOI:** 10.1007/s11356-024-32676-5

**Published:** 2024-03-27

**Authors:** Godwin Olasehinde-Williams

**Affiliations:** 1 Department of Economics, Istanbul Ticaret University, Istanbul, 34445 Turkey; 2 Nizami Gajanvi Research Center of Sustainable Development & Green Economy, Azerbaijan State University of Economics, Baku, Azerbaijan

**Keywords:** Carbon pricing, EU ETS, Eurozone, Macroeconomics, Panel data analysis

## Abstract

This article contributes to the carbon pricing debate by providing new evidence on the aggregate macroeconomic effect of the European Union Emissions Trading Scheme (EU ETS) in the Eurozone. To this end, a novel macroeconomic performance index is created to capture the overall economic performance of the Eurozone countries. The index is a weighted aggregation of key macroeconomic variables—GDP growth rate, inflation rate, employment rate, exchange rate, and long-term inflation rate—for the 19 member countries of the group. The effect of the EU ETS on this macroeconomic performance index is then empirically examined while controlling for the effects of physical capital accumulation, human capital accumulation and regulatory quality. A panel framework covering the period 2005–2022 is set up to achieve this objective and the relationship is examined using panel method of moments quantile regression with fixed effects, as well as fixed and random effects regressions of Driscoll and Kraay. A number of important revelations are made. Firstly, the behavior of the macroeconomic performance index constructed clearly reflects the economic reality of the Eurozone, with downward spikes visible in periods corresponding with the economic crisis of 2007–2009, the Eurozone debt crisis of 2010/2011, the aftermath of the financial crisis of 2016, and periods around the outbreak of the Coronavirus (COVID-19) pandemic. Hence, a case is made for the use of the macroeconomic performance index as a superior aggregate measure of the overall economic performance in empirical research. Secondly, a statistically significant negative effect of the EU ETS on aggregate macroeconomic performance in the Eurozone is confirmed. This shows that there are significant economic costs associated with the use of carbon pricing as a means of lowering pollution. Thirdly, the findings further show that the negative impact gradually decreases (in absolute values) from lower to higher quantiles. Overall, higher carbon prices cause greater economic disruption when macroeconomic performance is relatively poor but have less damaging effect when aggregate economic performance is relatively strong. Policy recommendations based on the study findings are also provided.

## Introduction

Given the urgent need to curtail greenhouse gas emissions and avert catastrophic climate consequences, the European Union (EU) has set ambitious targets in the recently introduced Fit-for-55 package (Riechmann et al. 2022; Olasehinde-Williams & Folorunsho 2023). The region intends to reduce emissions by 55%—relative to 1990 levels—by the year 2030 and subsequently achieve net zero emissions by the year 2050. Carbon pricing is one of the key measures being exploited by the Union in achieving the main objectives of the Fit-for-55 package. This is due to the internalization of the negative externality in market transactions through the imposition of carbon pricing being widely acknowledged as the most economically efficient means of dealing with pollution (OECD 2016). By incorporating costs associated with emissions, carbon pricing compels stakeholders to factor in the environmental consequences of their activities when making decisions. Carbon pricing typically takes the form of either emission taxes (see Brannlund & Persson 2012; Bumpus 2015; Dong et al. 2017) or emissions trading schemes (see Hopkin 2004; Heindl 2017; Perino & Pioch 2017). Regardless of the nature, however, carbon pricing generally provides incentives for emission reduction by assigning a monetary value to consumption and production emissions. Carbon pricing thus directly targets the carbon footprint of economies. Moreover, carbon pricing enhances long-term predictability, thus enabling stakeholders to make well-informed investment choices. Additionally, it establishes environmental certainty by imposing limits on emissions from different covered sources (Skjærseth & Eikeland 2013).

Although the EU is the third largest emitter after China and the USA, it took a while before carbon pricing in the form of emissions trading was embraced in the region due to the initial perception of it as a ‘license to pollute’ (Egenhofer 2007). It only received acceptance following the imposition in 1997 of the emissions trading provisions (Egenhofer 2003; Philibert & Reinaud 2004). However, since its adoption in Europe, carbon pricing in the form of emissions trading has continued to grow in importance. Currently, the EU emissions trading system (EU ETS) is undergoing reforms to enable the region to achieve the more stringent emissions reduction targets set for 2030 and 2050 (Barnes 2021). Consequently, carbon prices have reached unprecedented records in recent times in Europe.^1^ Efforts are also ongoing under the Energy Taxation Directive to align energy taxes with the ambitious emissions reduction targets of the region (Barnes 2021). Therefore, carbon pricing has clearly become an integral component of the broader climate policy architecture of the EU.

That carbon pricing significantly lowers emissions is not in doubt. It has been empirically established that a tightening of the carbon pricing regime elicits a strong and instantaneous rise in energy prices as well as a persistent decline in emissions (see Känzig 2021). This may however not come without an economic cost. For instance, higher carbon prices are likely to trigger price changes across various sectors in a manner that affects economic conditions. In the power sector of the region, a large chunk of electricity generation occurs through the burning of fossil fuels. Thus, movements in carbon prices are able to alter the cost of electricity generation from carbon-intensive technologies such as coal-fired plants. While the ultimate outcome may be a reduction in carbon emissions, it would also result in a higher cost of electricity that would be passed on to consumers (Wong & Zhang 2022). The transportation sector is also a major energy consumer, as well as a major contributor to carbon emissions (Danish et al. 2020). The transport sector in Europe is therefore also highly vulnerable to carbon price changes. Approximately 40% of energy consumption occurs in the transportation sector of the region, and 25.4% of total carbon emissions also occur in this sector (Tzeiranaki et al. 2023). With this degree of dependence on fossil fuels, it is to be expected that carbon price movements would be reflected in transportation costs faced by the public. The industrial sector in the European Union stands as the third largest energy consuming sector (25.6%).^2^ As such, industries pay close attention to changes in fuel prices due to its unique status as a major production input that can impact profitability (Ho et al. 2008). Eurozone countries where energy-intensive industries dominate are likely to experience greater economic decline in output, jobs, and profits as a result of higher energy prices induced by higher carbon prices (Ho et al. 2008). The residential and commercial buildings sector accounts for the largest share of energy consumption in the EU, majorly through electricity consumption and gas-burning furnaces. It goes without saying that carbon price changes would impact fossil fuel combustion costs in this sector.

Overall, higher energy prices directly drive headline inflation. Higher energy prices also indirectly drive core inflation through increases in the input prices for a range of goods that make up the core consumption basket (Daniel & Shamsfakhr 2022). Consumer prices rise and economic activities decline, consequently resulting in increased unemployment and lower output (Känzig 2021). Effects of carbon pricing in the Eurozone may thus be similar to those of a supply shock in the economy. Thus, as carbon prices rise in the Eurozone, key macroeconomic indices such as inflation, employment, and gross domestic product are likely to be adversely affected. Carbon pricing also creates the risk of “carbon leakage,” which could occur as a result of the relocation of carbon-intensive production processes to countries outside the region with less stringent environmental regulations or due to the replacement of EU goods with more carbon-intensive imports (Naegele & Zaklan 2019). Another concern about carbon pricing, therefore, is its potential to initiate cross-border externalities that result in trade and exchange rate distortions (Keen & Kotsogiannis 2014; McKibbin et al. 2014). On the positive side, however, higher carbon prices could improve the economy by contributing directly to government revenues. In general, how quickly carbon emissions fall is dependent on how quickly the economy is able to adjust to changes in carbon prices. Yet, at the moment, detailed knowledge backed by empirics on the economic effects of carbon pricing is still minimal. An understanding of the aggregate macroeconomic effect of carbon pricing is what this study provides. Such information is useful for policy decision-making geared towards the attainment of a balance between macroeconomic stability and climate action in Europe.

This study is an addition to the carbon pricing debate in the EU. Marginal contributions made by this paper to literature are as follows. Firstly, although carbon pricing is increasingly being researched, the focus of extant literature has so far been on its carbon reduction effectiveness (see Andersson 2019; Bureau et al. 2019; Guo et al. 2020; van den Bergh & Savin 2021; Lin & Huang 2022). Literature on the potential associated economic effects of the policy is still growing. This study expands the body of knowledge by exploring this area. Secondly, carbon pricing in the form of carbon taxes has been the most widely researched, whereas, carbon emissions trading is currently regarded as an alternative cost-effective carbon pricing approach towards emissions abatement (Andersen 2004; Wang et al. 2019; Runst & Thonipara 2020; Zhu et al. 2020). This study fills the gap by examining the economic by-effect of carbon emissions trading in the Eurozone. Thirdly, the larger proportion of studies to have considered the nexus between carbon pricing and the economy is focused on the microeconomic effects of the scheme, primarily because of the direct impact that carbon pricing has on firms. This study instead concentrates on the macroeconomic effect of the scheme.

Moreover, past studies considering the macroeconomic effects of carbon pricing have focused mainly on specific macroeconomic indexes such as economic growth, inflation, and unemployment (see among others, Driscoll 2020; Känzig 2021, 2023; Koh et al. 2021; Känzig & Konradt 2023). This study extends the literature by computing the macroeconomic performance index introduced by Ekren et al. (2017) for the 19 countries of the Eurozone. Empirical evidence shows that this aggregate index is a superior measure of the overall state of a nation’s economy. By aggregating changes in economic growth, inflation, unemployment, interest rate, and exchange rate, this index provides a holistic understanding of the economy. The effect of carbon pricing on this aggregate measure of economic status is examined for the first time in this study. Finally, this study also contributes to the body of knowledge on the empirical front. A battery of econometric techniques—panel method of moments quantile regression with fixed effects, as well as fixed and random effects regressions of Driscoll and Kraay—are utilized to ensure the robustness of the study outcomes. The panel method of moments quantile regression with fixed effects model is preferred in this study due to its ability to produce robust estimates in the face of endogeneity, heteroscedasticity, non-normality, skewness, and outliers. The fixed and random effects regressions of Driscoll and Kraay are specifically chosen due to their abilities to generate reliable results in the presence of spatial or cross-sectional dependence.

The remainder of this article proceeds in the following manner. In the next section, the study background is presented. In Sect. 3, related literature is reviewed. In Sect. 4, the theoretical framework and model are described. Section 5 explains the data and methodology employed. Section 6 shows and discusses the empirical findings. Section 7 concludes the discussion and presents policy recommendations.

## Background

### Command-and-control regulations

Command-and-control regulations are the traditional policy approaches for mitigating environmental pollution (Aldy & Stavins 2012; Wall 2022). These regulations are predominantly either technology-based or performance-based. The technology-based regulations generally enforce the use of specific types of equipment and techniques that are deemed more energy-efficient or eco-friendly. Performance-based regulations, on the other hand, simply specify permissible emission rates while allowing the regulated entities to determine their preferred methods of achieving the permitted level of emissions. As such, performance-based regulations exhibit greater flexibility than technology-based alternatives. However, although these technology and performance-based approaches are relatively effective for environmental protection, they result in non-cost-effective outcomes in which several firms end up using unduly expensive processes to lower their pollution (Aldy & Stavins 2012). These traditional approaches do not dynamically incentivize the adoption of processes that are environmentally and economically superior. On one hand, once regulated entities achieve a particular performance standard, there is no additional incentive for them to employ even more efficient processes. Technological standards, on the other hand, significantly inhibit innovation as regulated entities are limited in their choice of technology.

### Carbon pricing

To address the key weaknesses of the conventional command-and-control approach, market-based policy instruments mainly in the form of carbon pricing have been proposed. Carbon pricing is regarded as more cost-efficient than command-and-control (Harrington et al. 2004). Carbon pricing induces changes in production/consumption habits through the influence of the market forces (Metcalf 2007). Rather than imposing restrictions on emissions, carbon pricing incentivizes sustained environmental protection (Hahn & Stavins 2011). Carbon taxes are regarded as the simplest form of carbon pricing (Metcalf 2007). Carbon taxes are based on Pigou’s (1920) tax system, called Pigouvian taxes, in which prices are exogenously decided and markets are left to adjust accordingly to the price changes. With carbon taxes, policymakers are able to set tax rates in monetary values per ton of emissions. Economically, for such a tax to be cost-effective, it must cover all emission sources, and for it to be efficient, it must equal the marginal benefit of emission reduction (social cost of carbon) (Interagency Working Group on Social Cost of Carbon 2010). Carbon taxes establish relative certainty about the marginal cost of compliance and thus lower uncertainty about investment outcomes (Weitzman 1974).

Another form of carbon pricing gaining widespread popularity is the cap-and-trade systems. This alternative approach limits the aggregate amount of emissions generated by regulated sources (Goulder & Schein 2013). This is achieved through the creation of a limited quantity of tradable emission allowances that regulated sources are forced to give up to cover their emissions (Stavins 2007). Regulated entities are thus faced with the choice of either reducing their emissions or giving up their allowances. Regardless of how the allowances are distributed ab-initio, trading ensures the most efficient use of the allowances by covering the costliest emissions and incentivizing the least costly reductions (Montgomery 1972; Hahn & Stavins 2011). Thus, while carbon taxes require that a set emission price yields a quantity of emissions, cap-and-trade requires that a set aggregate quantity yields a price on emissions through trading. There are several elements of the cap-and-trade system that must be carefully considered and designed as part of the responsibilities of policymakers (Hussen 2012). First, the number of allowances to be given out must be determined. Second, the emission cap must be established. Third, types of emissions and sources to be covered must be identified. Finally, the mode of distribution of the allowances must also be determined.

### The European Union Emissions Trading Scheme (EU ETS)

The cap-and-trade system in the form of the EU ETS, which was launched in 2005, has become the cornerstone of Eurozone’s climate policy (Venmans 2012). Being the first and largest carbon market, it is arguably the most important carbon market in the world (Ellerman et al. 2016; Dou et al. 2022). At the moment, it covers 30 countries within Europe. It also covers approximately 40% of the EU’s total emissions. As a cap-and-trade system, EU ETS installations are required to give up as many allowances as they emit annually. Trading of allowances among operators is also freely permitted before the compliance date is reached. So far, the EU ETS implementation has been divided into three phases. In the first phase (2005–2007), the emissions cap was set at 2298 Mt CO2e per annum. The first phase was however characterized by an oversupply of allowances in which total allocation exceeded verified emissions (European University Institute 2019). The second, third, and fourth phases introduced in 2008–2012, 2013–2020, and 2021–2030 respectively improved on the first phase by permitting regulated entities to bank unused permits for later use. Borrowing against future emissions reductions was also introduced in the later phases. Moreover, the EU ETS coverage has been substantially widened in the latter phases, with previously unregulated sectors such as aluminum manufacture and aviation brought under regulation.

### Carbon pricing and the economy

While carbon pricing significantly succeeds in lowering emissions, its potential adverse economic consequences have however become an issue (see Baumol & Oates 1971; Pearce 1991; Pearce & Turner 1991; Goulder 1995; Koeppl et al. 1996; Andersen et al. 2006; Martin et al. 2014; Andersson 2019; Ellerman & Buchner 2008; Ellerman et al. 2010; Anderson et al. 2010). Concerns regarding the possible adverse effects of carbon pricing on key macroeconomic variables were registered right at the inception of the theoretical and political discourse on its use (Köppl & Schratzenstaller 2022). This apprehension contributed to the hesitancy exhibited by policymakers in adopting carbon pricing as it began to gain popularity (Köppl & Schratzenstaller 2022). In response to these concerns, the concept of the double dividend hypothesis emerged in literature. According to the hypothesis, it is possible to achieve both environmental and economic improvements through carbon pricing. Carbon prices can lower pollution (first dividend). It can also lower overall economic costs by financing reductions in preexisting taxes (second dividend). The general idea is thus that carbon pricing can bring about environmental and economic benefits simultaneously (Jaeger 2012).

## Empirical literature review

Extant literature on the link between EU ETS and the economy has been predominantly microeconomic in nature, focusing on indicators such as investment decisions, output, employment, revenue, and profit of firms. Wall (2022) attributes this to the fact that carbon emissions trading more directly affects firms and is thus more relevant to them. For instance, Anderson et al. (2010), in a study conducted on Irish firms over the period 2005–2007, show that EU ETS alters the investment decisions of firms. Jaraite-Kažukauske and Di Maria (2016) similarly find that the second phase of EU ETS altered the investment decision of Lithuanian firms. The conclusion reached by Commins et al. (2011) on the effect of EU ETS on European firms between 1996 and 2007 is that it lowers profits and productivity. The difference-in-difference study by Yu (2013) concludes that EU ETS subsequently led to a decline in the profitability of Swedish firms. The analysis of firm-level data for firms covered by EU ETS conducted by Dechezleprêtre et al. (2023) using the difference-in-difference technique however shows that the regulation positively impacted the revenues and assets of the firms. Also using the difference-in-difference approach, Marin et al. (2018) report that EU ETS improves investment and labor productivity. Studies by Martin et al. (2014), Lutz (2016), and Löschel et al. (2016) on German manufacturers likewise reveal that the first phase of EU ETS improved output and productivity. Studies emphasizing the ability of EU ETS to cause carbon leakage are also quite common. Several authors have documented how carbon trading schemes in the EU encourages firms to move their production to non-EU nations so as to be able to operate under more cost-efficient conditions (Jaffe et al. 1995; Levinson & Taylor 2008; Koch & Basse Mama 2016; De Beule et al. 2022).

Literature on the macroeconomic effect of EU ETS is however still limited. Only a handful of studies currently exist. Känzig (2021), with the aid of VAR analysis, examines the aggregate and distributional macroeconomic impacts of carbon policy shocks reflected in the EU ETS over the period 1999–2018. The study findings indicate that shocks that trigger a tightening of the carbon pricing regime raise energy prices, unemployment and consumer prices, as well as lower industrial production. The general conclusion is that carbon trading schemes affect economic activities in Europe. A similar study by Känzig and Konradt (2023) examines the effect of climate policy shocks on the macroeconomy of Europe. The study specifically considers the macroeconomic impacts of climate policy shocks reflected via the EU ETS between 1999 (introduction of the Euro) and 2019 (start of the COVID-19 pandemic). The study outcome similarly shows that climate policy shocks trigger increases in energy prices, headline consumer prices and unemployment, as well as decline in GDP and industrial production. McKibbin et al. (2014) also conclude that the sustained rise in general price levels caused by higher carbon prices is capable of causing a decline in economic activities and also triggering wage negotiations due to reduced purchasing power of workers. Wall (2022) carry out both cross-country and panel analyses on the investment and growth effects of the EU ETS over the period 1999–2012. While the study finds no significant effect on investment, the results show that EU ETS lowers economic growth in countries where the trading scheme is in use.

The overview of extant literature on the EU ETS-economy nexus provided clearly shows that studies focusing on the macroeconomic effects of carbon pricing are still limited in extant literature. Most related studies are focused on the microeconomic effects of the scheme. In addition, the handful of studies considering the macroeconomic effects focus mainly on specific macroeconomic indexes such as economic growth, inflation and unemployment. This study fills this identified gap in literature by computing an aggregate macroeconomic performance index for the 19 countries of the Eurozone. This aggregate measure of changes in economic growth, inflation, unemployment, interest rate, and exchange rate is a superior measure of the overall economic performance of nations. The effect of carbon pricing on this aggregate measure of economic status is examined for the first time in this study.

## Theoretical framework and model

Traditional economic growth theories identify physical capital accumulation and human capital accumulation as two key determinants of the economic status of nations (Mankiw et al. 1992; Nawaz et al. 2014). These two key determinants of macroeconomic performance therefore serve as the base regressors in the model used in this study. Moreover, good institutions are also identified in literature as sources of incentives that promote economic improvements (see North 1990; Hall & Jones 1999; Acemoglu & Robinson 2010; Ntom Udemba et al. 2022; Balcilar et al. 2020; Bekun et al. 2023). Institutional quality is therefore also included as one of the regressors in the empirical model. More specifically, as climate policy effectiveness is heavily dependent on the quality of policy enforcement, regulatory quality is used as the proxy for institutional quality. This is to account for country-specific variations in quality of regulations that can impact policy effectiveness. Finally, alongside these regressors established by economic theory and empirics as key determinants of economic performance, EU ETS is also included as a regressor.

Stemming from the above, the following level-log econometric model is specified:

1 $${MPI}_{{\text{it}}}={\upbeta }_{0}+{{\upbeta }_{1}{\text{LCP}}}_{{\text{it}}}+{\upbeta }_{2}L{INV}_{{\text{it}}}+{\upbeta }_{3}{{\text{LHC}}}_{{\text{it}}}+{\upbeta }_{4}{{\text{LRQ}}}_{{\text{it}}}+{\upvarepsilon }_{{\text{it}}}$$

In Eq. (1), *i* and *t* refer to the cross-sectional and time dimensions. The ${\beta }_{j}s$ are the parameter estimates of interest. ${\varepsilon }_{it}$ refers to the idiosyncratic error term. MPI refers to the macroeconomic performance index, which is the measure of aggregate economic performance. CP represents EU ETS, INV stands for physical capital accumulation, HC is human capital accumulation, and RQ is regulatory quality, the proxy for institutional quality. Logarithmic forms of carbon prices, physical capital accumulation, human capital accumulation, and regulatory quality are used for econometric analysis. As physical capital accumulation, human capital accumulation, and institutional quality are regarded as drivers of economic improvements, it is expected that ${\beta }_{2}$, ${\beta }_{3}$, and ${\beta }_{4}$ should all carry positive signs. If the argument made by this study is accurate, then it is expected that ${\beta }_{1}$ should display a negative sign, indicating that carbon pricing (EU ETS) worsens the aggregate economy of the Eurozone countries. The specified model in Eq. (1) is unique in the sense that it introduces the macroeconomic performance index as a novel aggregate measure of economic performance. Unlike past studies considering the economic effects of carbon pricing that have concentrated on specific macroeconomic indexes, this model provides a means of distinctly assessing the overall economic effect of carbon pricing in the Eurozone. Moreover, empirical evidence confirms that the economic performance assessment via such an aggregate measure is superior to the use of specific economic variables (Ekren et al. 2017). By aggregating changes in economic growth, inflation, unemployment, interest rate, and exchange rate, the outcome derived from this model is able to give a holistic understanding of the macroeconomic effect of EU ETS.

## Data and methodology

### Data

The macroeconomic performance index—the dependent variable—is first computed for the 19 countries of the Eurozone following the approach introduced by Ekren et al. (2017). The index is a weighted aggregation of the key macroeconomic variables—GDP growth rate, inflation rate, employment rate, exchange rate, and long-term interest rate. All the macroeconomic variables are used in their percentage forms. To prevent more volatile components from dominating the variability of the index, a variance-weighted scheme is employed. To this end, each variable is multiplied by the inverse of its variance for the sample period. Overall, the MPI index is mathematically obtained as follows:

2 $$MPI={w}_{y}\left({y}_{t}-{y}_{t-1}\right)+{w}_{emp}\left({emp}_{t}-{emp}_{t-1}\right)-{[w}_{\pi }\left({\pi }_{t}-{\pi }_{t-1}\right)+{w}_{i}\left({i}_{t}-{i}_{t-1}\right)+{w}_{exr}\left({exr}_{t}-{exr}_{t-1}\right)]$$

where *w* refers to the weight, *y* represents economic growth rate, *emp* stands for employment rate, *π* refers to inflation, *i* is interest rate, and *exr* is exchange rate.

In a simpler form, MPI could be mathematically represented as:

3 $$MPI=\Delta y+\Delta emp-(\Delta \pi +\Delta i+\Delta exr)$$

The regressor of interest—carbon emissions trading—is proxied with EU ETS. All member countries of the Eurozone are covered by the EU ETS. Being the first and largest carbon market, it is arguably the most important carbon market in the world (Ellerman et al. 2016; Dou et al. 2022). In addition, in line with traditional economic growth theories, the key determinants of economic performance of nations discussed in the previous subsection—physical capital accumulation, human capital accumulation, and institutional quality—are also included as controls.

For the empirical analysis, annual data on the 19 countries within the Eurozone over the period 2005–2022 is used. Data availability informs the choice of the sample period. EU ETS was introduced in the year 2005. Countries included in the analysis are as follows: Belgium, Germany, Ireland, Spain, France, Italy, Luxembourg, Netherland, Austria, Portugal, Finland, Greece, Slovenia, Cyprus, Estonia, Malta, Latvia, Lithuania, and Slovakia. These countries are the Eurozone member countries (countries that have adopted the Euro as their currency). The summary statistics of the variables are reported in Table 1. A look at the variables of interest (MPI and CP) shows that the computed MPI has an average index value of − 0.035 and ranges between − 2.161 and 6.451 with a standard deviation of 0.619. CP, on the other hand, ranges between 2.364 EUR and 81.345 EUR with an average value of 19.29 EUR and a standard deviation of 19.83. Moreover, the Jarque–Bera results indicate that all the variables are not normally distributed. The sources and measures of the variables are further summarized in Table 2.

**Table 1:** Statistical properties of variables

Variable	Mean	Max	Min	Std. dev	Skew	Kurt	J-B
MPI	− 0.035	6.451	− 2.161	0.619	3.856	41.056	20292.06***
CP	19.290	81.345	2.364	19.830	2.073	6.764	23.531***
INV	1.310	07.660	0.002	1.930	1.809	5.160	239.102***
HC	102.150	118.081	96.553	3.250	1.637	8.215	481.945***
RQ	1.238	2.045	0.144	0.388	− 0.078	2.251	7.866**

(1) Skew, Kurt, and J-B denote skewness, kurtosis, and the statistics of the Jarque–Bera test for normality (2) ***, **, and * denote statistical significance at 1%, 5%, and 10%, respectively

**Table 2:** Description of variables

Name of variable	Abbreviation	Scale of measurement	Source
GDP growth rate	Y	Annual percentage	World Development Indicators, 2023
Employment rate	Emp	Annual percentage of total labor force	World Development Indicators, 2023
Inflation rate	$\pi$	Annual percentage change in consumer prices	World Development Indicators, 2023
Exchange rate	Exr	Percentage change in local currency per US dollar	World Development Indicators, 2023/author’s calculation
Interest rate	I	Annual percentage	European Central Bank Statistics, 2023
Macroeconomic Performance Index	MPI	Index	Author’s calculation
Carbon emissions trading	CP	Euro	ICE Carbon Futures Indices, 2023
Physical capital accumulation	INV	US dollars	World Development Indicators, 2023
Human capital accumulation	HC	US dollars	World Development Indicators, 2023
Regulatory quality	RQ	Index	World Governance Indicators, 2023

### Methodology

#### Selection of panel data estimation approach

Data sequences used for empirical analysis are made up of a relatively small N (19 cross sections) and a relatively small T (18 years). Also, N is greater than T (19 > 18). In practice, this shows that the time dimension is not long enough to support time-series regression for each cross section. It also shows that N is not large enough to support the averaging across cross sections required to generate consistent outcomes and ensure the validity of the central limit theorem. These indicate that the data series for this study are not large enough to be treated as panel time-series; consequently, a traditional panel data analysis procedure is followed. Specifically, fixed and random effects-based techniques are employed. The process of selecting the most appropriate traditional panel analysis procedure involves various tests and considerations. To this end, the Hausman (1978) test is first conducted to determine the most suitable between the fixed and random effects estimators for the specified model. The null hypothesis of the Hausman (1978) states that no correlation exists between the error terms and the regressors, the rejection of which indicates the superiority of the fixed effects method.

#### Examination of fixed effects model assumptions

Based on the Hausman (1978) test outcome, Eq. (1) is estimated with the fixed effects estimator. As such, key assumptions about the error terms (heteroscedasticity, serial correlation and cross-sectional dependence) are checked for validity. The modified Wald test is also performed to check for possible groupwise heteroscedasticity in the residuals of the selected model. The null hypothesis for the Wald test is that the error terms have constant variances across panel units. To check for serial correlation, the Wooldridge (2002) test for autocorrelation is employed. The test is capable of detecting first-order serial correlation in the errors of panel models. It is also more flexible with regard to assumptions on the behavior of the heterogeneous individual effects. The null hypothesis in this case is that there is no serial correlation in the specification. Finally, the Pesaran (2021) cross-sectional dependence test is performed to determine whether substantial cross-sectional dependence exists in the errors. This dependence may result from common shocks, unobserved components that end up as parts of the error term, spatial dependence, and idiosyncratic pairwise dependence in the disturbances. The choice of this particular test is informed by its suitability for cases where cross sections are greater than the time dimension (N > T). Specifically, two variants of the fixed effect technique that are able to, between them, handle all the aforementioned challenges, along with several others are used—the panel method of moments quantile regression with fixed effects and the fixed effect regression of Driscoll and Kraay.

#### Panel method of moments quantile regression (MMQR)

First, the MMQR with fixed effects of Machado and Silva (2019) is performed in establishing the effect of the carbon trading scheme (EU ETS) on the aggregate macroeconomic performance of the Eurozone while controlling for the impacts of physical capital, human capital, and regulation quality. This variant of the traditional fixed effect model is preferred to available alternatives due to its ability to produce robust estimates in the face of heteroscedasticity, non-normality, skewness, outliers, and endogeneity (Afshan et al. 2022; Gimenes & Guerre 2022; Lee et al. 2022). The method’s ability to handle endogeneity is quite important for this study as Känzig and Konradt (2023) argue that economic outcomes are able to compel the government to adjust carbon prices or delay planned price alterations. In addition, the identification of nonnormality in the underlying data series (see Table 1) provides a justification for the use of MMQR, as the technique is robust to this challenge. Also, the technique provides an avenue for observing distributional effects across quantiles since it is able to segregate the distributive effect of the regressors on the regressand across quantiles. The MMQR technique is thus suitable for analyzing nonlinear relations. This is especially important as Ekren et al. (2017) state that the macroeconomic performance index exhibits a nonlinear structure.

Panel MMQR with fixed effects equation is specified as follows:

4 $${Q}_{Y}\left(\tau \left|{X}_{it}\right.\right)=[{\alpha }_{i}+{\delta }_{i}q]+{{Z}^{\prime}}_{it}\beta +{{X}^{\prime}}_{it}\gamma q$$

${Q}_{{\text{Y}}}\left(\tau |{X}_{it}\right)$ = quantile distribution of the dependent variable MPI, which is conditional upon the location of the independent variables. ${{X}^{\prime}}_{it}$ = vector of independent variables $\left(LCP{I}_{it},LIN{V}_{it},LR{Q}_{it}\right)$. ${\alpha }_{i}\left(\tau \right)\equiv$ ${\alpha }_{i}+{\delta }_{i}q\left(\tau \right)$ = scalar coefficient representing the quantile-τ fixed effect for individual *i*. ${{Z}^{\prime}}_{it}$ = vector of known differentiable transformations of the components of ${{X}^{\prime}}_{it}$. ${\left[{\alpha }^{\prime}, \beta , \delta ,{\gamma }^{\prime}, and\, q\left(\tau \right)\right]}^{\prime}=$ vector of parameters of interest.

#### Fixed effect regression of Driscoll and Kraay

Another potential problem in panel data analysis is the presence of spatial or cross-sectional dependence. This is especially common in panel data sets, such as the one used in this study, with cross sections that are not randomly sampled or in which common shocks simultaneously affect the cross sections (see Driscoll & Kraay 1998; Eberhardt et al. 2013; Olanipekun & Olasehinde‐Williams 2022; Olasehinde-Williams & Bekun 2024). While dependence of this nature does not affect the consistency of parameter estimates, it does distort the standard errors of the parameter estimates (Driscoll & Kraay 1998). Equation (1) is therefore again estimated with the fixed effects (within) regression model proposed by Driscoll and Kraay (DK-FE 1998).^3^ In addition to its ability to handle spatial/cross-sectional dependence, the method also generates standard errors that are robust to heteroscedasticity and serial correlation (Lee & Olasehinde‐Williams 2024). The fixed effects estimator is executed in two steps. In the first, a within-transformation of all the variables is performed in the following manner;

5 $${\widetilde{W}}_{it}={W}_{it}+{\overline{W} }_{i}+\overline{\overline{W}}$$

where *W*_*it*_ ϵ {*Y*_*it*_, *X*_*it*_}, ${\overline{W} }_{i}= {T}_{i}^{-1}\sum_{{t=t}_{i1}}^{{T}_{i}}{W}_{it}$,$\overline{\overline{W}}= {\left({\sum T}_{i}\right)}^{-1}\sum_{i}\sum_{i}{W}_{it}$, ${W}_{it}=$ vector of variables.

As the within-estimator corresponds with the ordinary least squares (OLS) estimator of Eq. (6), the equation is estimated via the pooled OLS with Driscoll and Kraay standard errors.

6 $${\widetilde{y}}_{it}={\widetilde{x}}_{it}^{\prime}\theta +{\widetilde{\varepsilon }}_{it}$$

${\widetilde{y}}_{it}$ and ${\widetilde{x}}_{it}^{\prime}$ are the transformed variables and ${\widetilde{\varepsilon }}_{it}$ is the transformed error term.

## Presentation and discussion of results

**Fig. 1:**
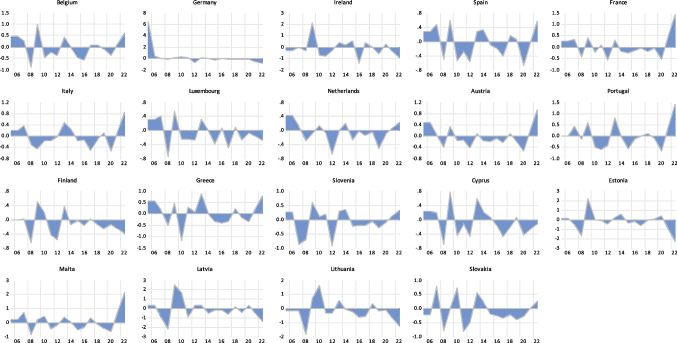
Area plots of the country-specific macroeconomic performance indices

The country-specific area plots of the macroeconomic performance index constructed for the 19 Eurozone countries are first presented in Fig. 1. The objective here is to examine how close to the economic reality of the Eurozone the index is. In addition to the volatility displayed, the graphs predominantly reflect downturns in the macroeconomic performance of the Eurozone in periods corresponding to major economic/financial crises in the Eurozone. Downturns are visible in periods around the global economic crisis of 2007–2009, the Eurozone debt crisis of 2010/2011, the aftermath of the financial crisis of 2016, and periods around the outbreak of the COVID-19 pandemic. Periods of economic recovery following these downturns are also visible from the graphs. Overall, the macroeconomic performance index constructed is a true reflection of the economic reality of the Eurozone.

**Fig. 2:**
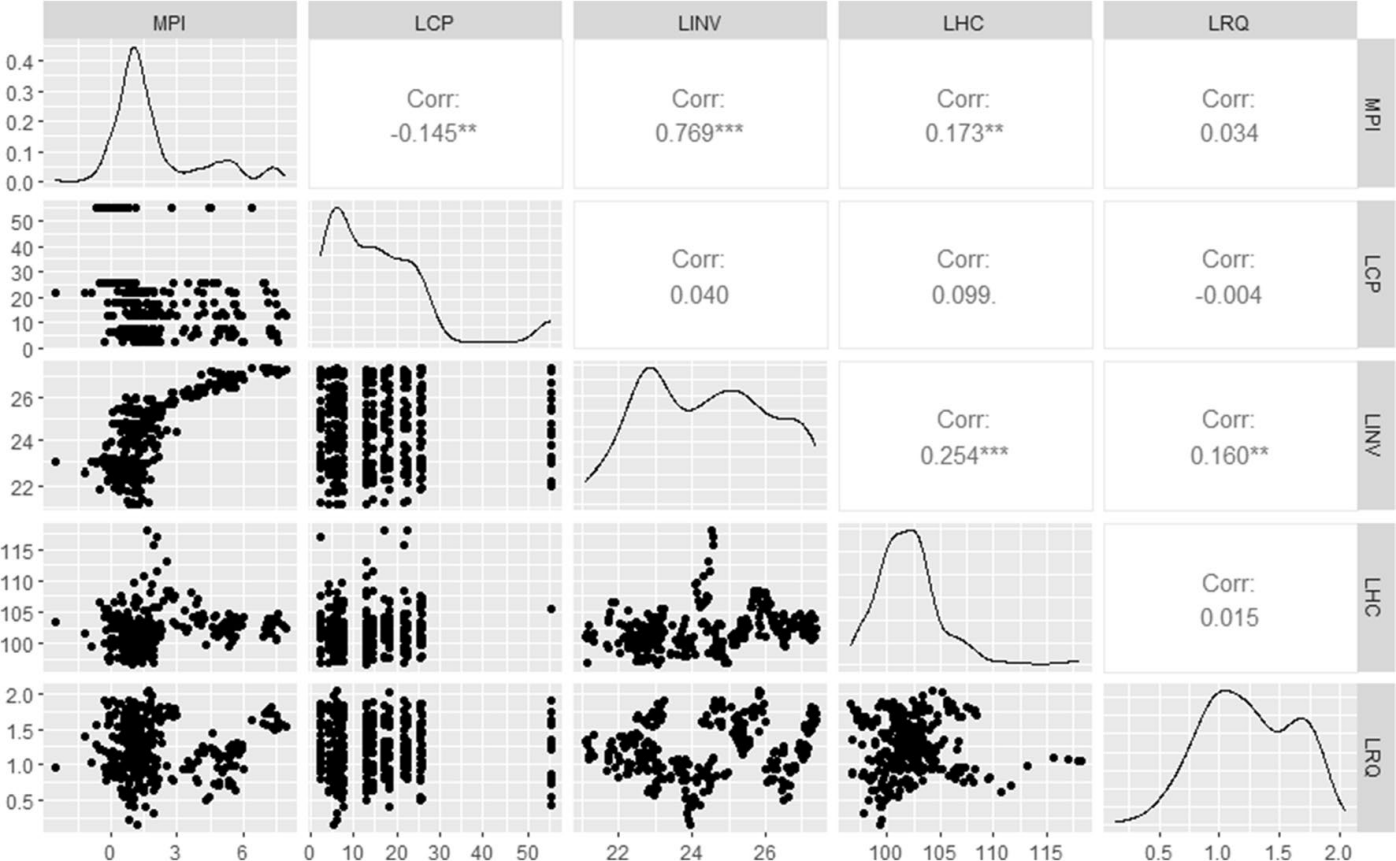
Correlation matrix and plots

Next, correlation plots are reported for two primary reasons. Firstly, these plots are utilized to establish the nature, strength and significance of the relationships between the variables incorporated in the specified regression equation. Secondly, these plots are used to test for the presence of multicollinearity among the regressors in the regression equation. Correlation coefficients exceeding 0.7 generally indicate a high linear relationship between the variables (Olasehinde-Williams 2023). As shown in Fig. 2, a statistically significant negative correlation exists between carbon prices and the macroeconomic performance index. This is the first inference that increases in carbon prices lower macroeconomic performance in the Eurozone and vice versa. Positive and significant coefficients are reported for the correlations between the macroeconomic performance index and the control variables—investment and human capital. The positive correlation between macroeconomic performance index and regulatory quality is also positive but insignificant. Regarding multicollinearity, all correlation values between the regressors are well below the threshold of 0.7. Moreover, multicollinearity is further formally tested using a variance inflation factor test for panel data. The centered variance inflation factor values reported in Table 3 are all well below the threshold of 5. Therefore, on the basis of both the correlation plots and the variance inflation factor, it can be concluded that multicollinearity is not a concern in this study.

**Table 3:** Variance inflation factor

Regressors	Centered VIF
LCP	1.064
LINV	1.029
LHC	1.038
LRQ	1.058

In Table 4, the result of the Hausman (1978) test conducted to determine the most suitable between the fixed effects and the random effects estimators is first reported. The relatively high and statistically significant test statistic reported indicates that outcomes generated by the fixed effects specification are likely to be superior. Decision is therefore made in favor of the fixed effects alternative in estimating the effect of carbon pricing on the aggregate macroeconomic performance. Next, the statistically significant test statistic for the modified Wald test shows that heteroscedasticity is a concern in this study. Lastly, the significant statistic for the Wooldridge (2002) test of serial correlation also indicates that serial correlation needs to be addressed in this study. These outcomes not only justify the use of the fixed effects technique, but also justify the variants used—panel method of moments quantile regression with fixed effects, as well as fixed and random effects regressions of Driscoll and Kraay. Between them, these methods are able to deal with the identified challenges.

**Table 4:** Panel data model selection test

Test	Test statistic	*P*-value
Hausman test for selecting between fixed and random effects	5.44^c^	0.066
Modified Wald test for heteroscedasticity	282.89^a^	0.000
Wooldridge’s test for serial correlation	13.009^a^	0.002

(1)^a^ and^c^ denote statistical significance at 1% and 10%, respectively

Given that the Eurozone countries are not randomly selected, and that common economic shocks can influence the economies of the Eurozone countries simultaneously, there is the likelihood that cross-sectional dependence would be a challenge in this study. Furthermore, cross-sectional dependence may occur due to proximity and spillover effects across the Eurozone countries. The Pesaran (2021) test to detect cross-sectional dependence is thus conducted and reported in Table 5. As shown in Table 5, the outcomes of the test confirm the presence of cross-sectional dependence in all the variables, except human capital. This finding provides a justification for carrying out additional regressions using the Driscoll and Kraay standard errors.

**Table 5:** Cross-sectional dependence test

	MPI	LCP	LINV	LHC	LRQ
Pesaran CD test	35.361^a^	55.479^a^	22.673^a^	− 0.460	16.756^a^
	(0.000)	(0.000)	(0.000)	(0.645)	(0.000)

(1) ^a^ denotes statistical significance at 1%. (2) *P*-values are in parentheses.

The conventional approach is that if T > N or if N > T but T is sufficiently large (generally 30 or above), testing the presence of unit root and consequently cointegration is required (see Baltagi & Baltagi 2008). Although for this study, N > T, the difference is however quite small as N is 19 and T is 18; unit root and cointegration testing are therefore still carried out to ensure that all bases are covered. Testing for stationarity is usually required to ascertain the integration properties of the data series (Bekun 2022). In Table 6, unit results obtained from the Im et al. (2003) as well as the cross-sectionally augmented IPS (Im et al. 2003) are reported. All variables exhibit nonstationarity in their level forms but exhibit stationarity in their differenced forms. Consequently, the Westerlund (2007) panel cointegration tests are performed. As reported in Table 7, both of the panel test statistics and one of the two group-mean test statistics confirm that the variables are cointegrated. The existence of a long-term relationship among the variables is thus established.

**Table 6:** Unit root test results

		Constant		Constant & Trend	
Variables	Tests	I_0_	I_1_	I_0_	I_1_
MPI	IPS	3.510	− 16.014^a^	− 0.712	− 12.704^a^
	CIPS	− 1.462	− 4.067^a^	− 1.929	− 3.962^a^
LCP	IPS	6.060	6.915^a^	16.771	− 13.182^a^
	CIPS	2.610	− 2.153^c^	1.700	− 2.661^c^
LINV	IPS	− 1.056	− 10.788^a^	0.465	− 11.239^a^
	CIPS	− 1.572	− 3.429^a^	− 2.616	− 3.487^a^
LHC	IPS	− 1.112	− 7.030^a^	− 0.402	− 6.316^a^
	CIPS	− 1.942	− 3.201^a^	− 2.304	− 3.611^a^
LRQ	IPS	− 0.664	− 13.715^a^	− 0.550	− 11.581^a^
	CIPS	− 2.050	− 4.822^a^	− 2.208	− 4.602^a^

(1)^a^ and^c^ denote statistical significance at 1% and 10%, respectively. (2) I_0_ and I_1_ represent level and first difference, respectively

**Table 7:** Panel cointegration test results

Statistic	Value	*Z*-value	*P*-value
*G*_t_	− 1.562	− 2.454^a^	0.007
*G*_a_	− 4.086	− 0.272	0.393
*P*_t_	− 7.258	− 4.312^a^	0.000
*P*_a_	− 4.272	− 4.886^a^	0.000

^a^Statistical significance at 1%

**Fig. 3:**
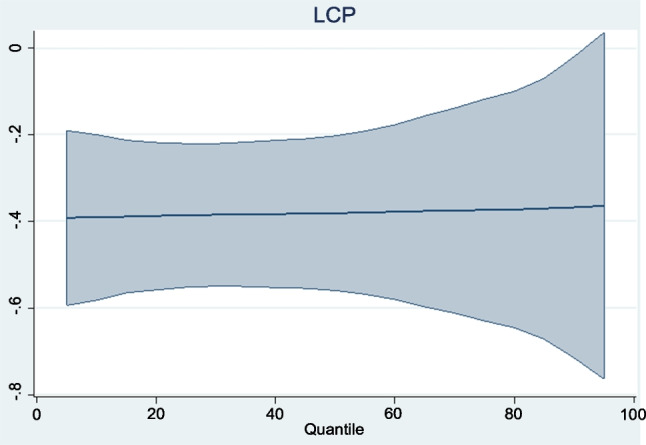
Trend of LCP coefficients across quantiles

**Fig. 4:**
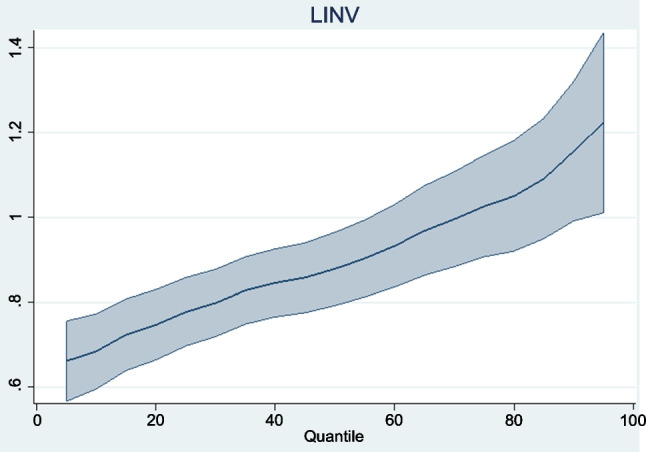
Trend of LINV coefficients across quantiles

**Fig. 5:**
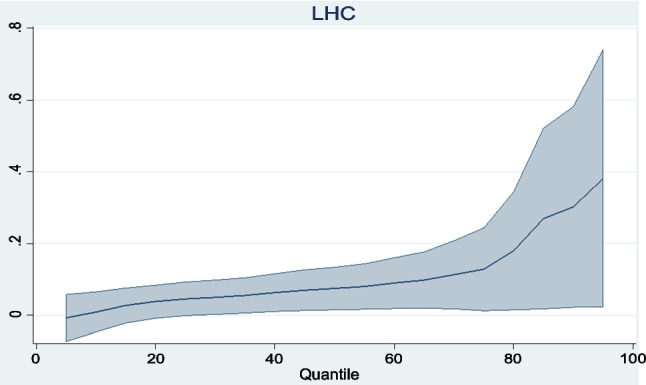
Trend of LHC coefficients across quantiles

**Fig. 6:**
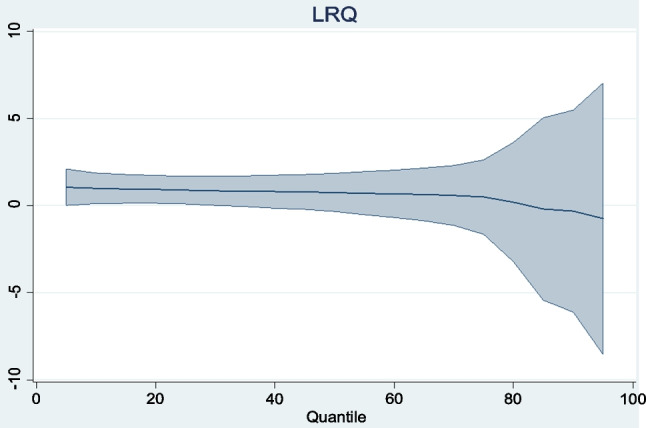
Trend of LRQ coefficients across quantiles

The panel regression results obtained are reported in Table 8. Graphical representations of the trends of the regressors across quantiles are also presented in Figs. 3, 4, 5, 6. To begin with, the coefficient of carbon price reported under the location parameters column shows that the marginal impact of carbon prices on the aggregate macroeconomic performance of the Eurozone is negative and statistically significant. It is worthy of mention that coefficients reported under the location parameters column are equivalent to the standard fixed effects results. To control for the distorting effect of cross-sectional dependence, coefficients from the fixed effects and random effects with Driscoll and Kraay standard errors (DK-FE and DK-RE) are also reported. The outcomes are not too different from what was obtained under the location parameters. The statistically significant negative effect of carbon prices on aggregate macroeconomic performance in the Eurozone is again confirmed. All three results indicate that a percentage rise in carbon prices can lower the overall quality of the macro economy by between 0.324 and 0.380%. By establishing the adverse impact of the carbon trading scheme in Europe on aggregate macroeconomic performance, this study extends the conclusions of Känzig (2021, 2023) and Känzig and Konradt (2023). Känzig (2021, 2023) concludes that carbon price regime tightening raises unemployment, lowers output and has inflationary impacts in Europe. This study confirms the adverse effect of EU ETS on three of the variables aggregated in the macroeconomic performance index individually. In a similar vein, Känzig and Konradt (2023) conclude that changes in the EU ETS shocks raise energy prices, inflation, unemployment and lower industrial production and GDP individually.

**Table 8:** Panel regression results

					MMQR quantiles				
	Location Parameters	Scale parameters	DK-FE	DK-RE	0.1	0.3	0.5	0.7	0.9
LCP	− 0.380^a^	0.007	− 0.380^b^	− 0.324^b^	− 0.391^a^	− 0.385^a^	− 0.381^a^	− 0.375^a^	− 0.367^b^
	(0.000)	(0.909)	(0.030)	(0.028)	(0.000)	(0.000)	(0.000)	(0.002)	(0.038)
LINV	0.900^a^	0.141^a^	0.900^a^	0.073^c^	0.684^a^	0.798^a^	0.900^a^	0.996^a^	1.155^a^
	(0.000)	(0.000)	(0.000)	(0.053)	(0.000)	(0.042)	(0.091)	(0.342)	(0.000)
LHC	0.075^b^	0.086^c^	0.114^a^	0.116^c^	0.010	0.051^b^	0.075^b^	0.113^b^	0.302^b^
	(0.015)	(0.062)	(0.000)	(0.057)	(0.735)	(0.036)	(0.013)	(0.019)	(0.034)
LRQ	0.753	− 0.406	0.216	0.494	0.990^b^	0.854^b^	0.753	0.587	0.319
	(0.179)	(0.675)	(0.231)	(0.103)	(0.027)	(0.048)	(0.177)	(0.504)	(0.914)

(1)^a^,^b^, and^c^ denote statistical significance at 1%, 5%, and 10%, respectively. (2) *P*-values are in parentheses. (3) DK-FE and DK-RE represent fixed effects and random effects with Driscoll and Kraay standard errors

However, coefficients reported under the location parameters, DK-FE and DK-RE, are average values of a scale outcome. The statistically significant values associated with investment and regulatory quality in the scale parameters column are indicative of the fact that the data is heterogeneous in nature. This identified heterogeneity confirms the need for the MMQR method. Based on the MMQR results, the impact of carbon prices on the overall macroeconomic performance of the Eurozone is negative and statistically significant across all quantiles and ranges between 0.367 and 0.391%. The results show that the negative impact gradually decreases (in absolute values) from lower to higher quantiles. This leads to the conclusion that higher carbon prices cause greater economic disruption when macroeconomic performance is relatively poor but has less damaging effect when aggregate economic performance is relatively strong. This is an indication that factors such as policy choices and economic situations may affect the strength of the impact of carbon pricing on the macroeconomic performance of the Eurozone. This outcome therefore confirms the claims made by Saqib and Dincă (2023) and Saqib et al. (2024) that countries generally require strategies that effectively manage the trade-off between macroeconomic performance and environmental sustainability.

The control variables—investment and human capital—have the expected significant positive signs under the location parameters, DK-FE and DK-RE. In line with economic theory and empirics, improvements in both variables thus induce improvements in the overall economic performance of the Eurozone. These findings are in accordance with economic theory and empirics, as discussed by Mankiw et al. (1992) and Nawaz et al. (2014). Further revelations from the MMQR results across quantiles show that the coefficients of investment and human capital exhibit gradual increases from lower to higher quantiles. These indicate that the human and physical capitals are relatively more effective in Eurozone countries with superior economic organization. With regard to regulatory quality, its impact on macroeconomic performance in the Eurozone, though positive, is statistically insignificant. No inference is thus made about its overall economic impact.

## Conclusion

There is undoubtedly a consensus among stakeholders that carbon pricing is the most economically efficient means of internalizing the negative environmental externalities of fossil fuel burning in the form of pollution. Empirical evidence detailing the unintended macroeconomic consequences of this market-based policy is however still sparse. This article contributes to the carbon pricing-macroeconomy nexus debate by providing new evidence on the aggregate macroeconomic effect of EU ETS in the Eurozone.

The macroeconomic performance index is first created as a weighted aggregation of key macroeconomic variables—GDP growth rate, inflation rate, employment rate, exchange rate, and long-term inflation rate—for the 19 countries of the Eurozone. Using a panel framework made up of 19 countries of the Eurozone for the period 2006–2020, panel method of moments quantile regression with fixed effects, as well as fixed and random effects regressions of Driscoll and Kraay are then performed.

A number of important revelations are made in the empirical analysis. An inspection of the graphs of the constructed macroeconomic performance index shows that it is a true reflection of the economic reality of the Eurozone. Downturns in periods corresponding to major economic/financial crises (global economic crisis of 2007–2009, the Eurozone debt crisis of 2010/2011, the aftermath of the financial crisis of 2016, and periods around the outbreak of the COVID-19 pandemic) in the Eurozone are visible. Spikes are also visible in periods of recoveries from these crises. Hence, this study makes a case for the use of the macroeconomic performance index as a superior aggregate measure of the overall economic performance in empirical research.

A statistically significant negative effect of carbon prices on aggregate macroeconomic performance in the Eurozone is confirmed. The findings further show that the negative impact gradually decreases (in absolute values) from lower to higher quantiles. Overall, higher carbon prices cause greater economic disruption when macroeconomic performance is relatively poor, but has less damaging effect when aggregate economic performance is relatively strong. This indicates that the economically stronger members of the Eurozone are likely to be less affected by higher carbon prices than the weaker ones. The distributional macroeconomic effect of carbon trading thus shows that the poorer countries of the Eurozone are more disadvantaged than the richer ones. An important implication of this outcome is that the less developed countries within the zone may choose to prioritize investment inflows without looking too closely at the eco-friendliness of the production processes involved since they are relatively less affected by the adverse effects of carbon pricing. Therefore, the cross-country variations in carbon pricing may be another justification for the pollution haven hypothesis.

Based on the study findings, several recommendations are put forward. For one, while designing carbon trading schemes, policymakers/governments should be aware of the potential negative aggregate macroeconomic effects. As this study shows, this fear is legitimate and should be properly factored into policy decisions about the unconventional energy market. Thus, changes to carbon prices must take into account the prevalent socio-economic conditions. Carefully designed measures to counterbalance the adverse macroeconomic effects of higher carbon prices should be adopted. For instance, the macroeconomic impact of carbon pricing will depend in part on the use of the revenue generated from the policy implementation. Thus, efficient recycling of revenues generated from carbon pricing should be a priority. Such revenues may be used to cushion the adverse effects among the most vulnerable groups within the economy.

Recycling schemes could also be used to incentivize research and development of less-emitting technologies that are relatively affordable. Such innovation and technological progress can ensure that the distortionary effect of carbon pricing is lowered without having to resort to a degrowth strategy, thus ensuring a sustainable transition to a low-carbon economy. Regulations should also be put in place to prevent the more advanced economies from taking advantage of the weaker countries by relocating dirty processes within their borders. In addition, the less developed economies of the Eurozone should be more strategic with the use of carbon trading schemes as their economies are more sensitive to carbon price movements. This likelihood that carbon pricing would cause carbon leakage in the Eurozone creates a need for international coordination and cooperation with regard to the use of the policy.

It is important to mention some caveats. This study acknowledges that the economic impact of carbon pricing is generally sector-specific, affecting some segments of the economy more than others. The economic impact likewise generally varies among groups within the economy, impacting some more than others. Furthermore, the effects of different carbon pricing methods are also likely to differ in terms of economic impact. These distributional impacts of different types of carbon pricing methods in the Eurozone are however not taken into consideration as the main focus of this study is to establish the aggregate macroeconomic consequences of carbon pricing in the Eurozone. Also, EU ETS has gone through a number of phases during which adjustments have been made to general regulations and coverage. There is the possibility that the macroeconomic consequences of the scheme may differ across phases. As such, this study may be extended in the future to cover specific sectors, specific countries, alternative carbon pricing methods, alternative environmental policies, and longitudinal studies to measure long-term implications.

## Data Availability

Data used for empirical analysis can be obtained from the author upon reasonable request.

## Footnotes

1 https://www.worldbank.org/en/news/press-release/2023/05/23/record-high-revenues-from-global-carbon-pricing-near-100-billion

2 https://ec.europa.eu/eurostat/statistics-explained/index.php?title=Final_energy_consumption_in_industry_-_detailed_statistics

3 While the Driscoll and Kraay standard errors are originally proposed for the fixed effects panel regression, statistical advancement has made it possible to also estimate random effects regression models (Generalized Least Squares only). Both results are therefore reported.
